# A Temple of Harmony

**Published:** 1921-06-18

**Authors:** 


### THE MATRONS' AND SISTERS' DEPARTMENT.

### A TEMPLE OF HARMONY.

In his beautiful vision of human existence under the image of "A Midsummer Night's Dream" Shakespeare brought upon his scene a group of men and women wandering, lost and bewildered, in the mazes of a wood, seduced by subtle poison into causeless strife, filled with empty passion, railing on those they should have reverenced, impatient, unjust, most wretched. Amid this darkness of material things no way out was to be found, no hope of ending an enmity as unreal as it was mischievous. Then came the dawn. One by one the sleepers, touched by the sun's rays, opened their eyes and stood gazing once more in kindly fashion on each other. "Are you sure," said one, "that we are awake? It seems to me that yet we sleep, we dream." But another bade them "follow to the temple." And so, moving forward together, the truth was borne in upon them—"Why, then, we are awake." Jealousy and discord had vanished. The bad dream was ended. The sun was up.

Some such thoughts as these we may be pardoned for entertaining in view of the proceedings last Friday at the opening of the General Nursing Council Headquarters at 12 York Gate, Regent's Park. A representative gathering assembled, and the names of men and women who strove long and steadfastly for the boon now within reach were brought to mind. It was a moving occasion for all in whom the divine gift of imagination has free play. Princess Christian's short address was admirable. In congratulating the nursing profession on the recognition and protection afforded by the Registration Act Her Royal Highness laid stress on the fact that the Council is representative of all parties, and expressed her confidence that all will join hands in one common endeavour to make the Council offices a temple of harmony and peace from which a great and beneficent influence may flow.

The nursing profession is conscious at last that it is awake. A great future, which none can guess, lies before it. Its leaders need every ounce of the energy with which their vocation endows them to compass the work which lies ahead. The next few years must witness a strenuous campaign to draw in every institution capable of taking a part, whether great or very small, in the business of fitting women to be nurses. First among the lessons taught in the probationary period must be the need for nurses to show a united front to the world by accepting universally the mingled privileges and responsibilities of registration. Unless registration be all-embracing it will, in the end, come to be written down a failure.

Up till now no voluntary State registration scheme has ever succeeded in attaining more than partial success. British nurses will not be satisfied with this. A long pull, a strong pull, and a pull all together can alone justify the hopes which have been entertained on the subject of State Registration. The proceedings on Friday, June 10, gave good hopes of their fruition.

